# STRENGTH OF ATTACHMENT TO RELIGIOUS BELIEFS DECREASES NEGATIVE ATTITUDES TOWARD OLDER ADULTS

**DOI:** 10.1093/geroni/igae098.2556

**Published:** 2024-12-31

**Authors:** Jessica Strong

**Affiliations:** University of Prince Edward Island, Charlottetown, Prince Edward Island, Canada

## Abstract

Ageism, a term identified by Butler in the 1960s, refers to any discrimination towards an individual based on their age: implicit, explicit, or internalized. The impacts of experiencing ageism are far reaching, including negative impacts on memory, mood, physical health, and even mortality. Two recent studies (Neto et al., 2023; Kim & Jung, 2020) investigated the protective role religiosity may have on attitudes towards older adults. These studies provided preliminary evidence that groups or cultures with stronger religious beliefs had better attitudes towards older adults. The current study examined the impact of attachment to a belief system and death anxiety on ageism in a population of adults (21-73 years old, N=31). Participants reported a variety of belief systems, including Christian, Buddhist, Muslim, and Atheist/Agnostic, and were separated into two groups based on the strength of their beliefs (attached or unattached). ANCOVA analyses showed that while holding death anxiety constant, there was no impact of religious attachment on positive ageism (F(1,14)=0.98, p=0.34, partial eta2=0.06), there was an impact of religious attachment on negative ageism (F(1,14)=4.52, p=0.05, partial eta2=.24), and belief that lives of older adults should be restricted ((F(1,14)=5.24, p=0.045, partial eta2=.34), both with large effect sizes. Although the sample was small and likely underpowered, the effect sizes suggest more research in this area is warranted. The sample was diverse in terms of belief systems and the results are discussed in the context of strength of belief system, including atheism or agnosticism.

